# Supplementary material for: Rhodococcus Strains from the Specialized Collection of Alkanotrophs for Biodegradation of Aromatic Compounds
Source: Molecules. 2023 Mar 5;28(5):2393. doi: 10.3390/molecules28052393 (PMC10005059; doi:10.3390/molecules28052393)
Supplement: Supplementary file 1 [file molecules-28-02393-s001.zip › molecules-2248040-SI.pdf]

Table S1 – *Rhodococcus* strains (http://www.iegmcol.ru)

Strain	Isolation source
R. aetherivorans	
IEGM 1250	Soil, Russia
R. erythropolis	
IEGM 190	Water, the Ob river, Tyumen region, Russia
IEGM 201	Water, spring, Olkhovski oil-extracting enterprise, Perm region, Russia
IEGM 202	Water, spring, Yarino-Kamenolozhski oil-extracting enterprise, Perm region, Russia
IEGM 203	Water, observing hydro-geological well, Yurchukski oil-extracting enterprise, Perm region, Russia
IEGM 204	Water, observing hydro-geological well, Unvinski oil-extracting enterprise, Perm region, Russia
IEGM 212	Sewage, Kharbin, China
IEGM 214	Sewage, Kharbin, China
IEGM 215	Mineral water, Surgut district, Tyumen region, Russia
IEGM 216	Oil-polluted water, Kama river reservoir, Perm region, Russia
IEGM 244	Municipal wastewater, Harbin, China
IEGM 250	Snow, Yakutia, Saha Republic, Russia
IEGM 251	Snow, Yakutia, Saha Republic, Russia
IEGM 252	Snow, Yakutia, Saha Republic, Russia
IEGM 265	Oil-polluted water, oil-extracting enterprise, Perm region, Russia
IEGM 266	Oil-polluted soil, oil-extracting enterprise, Perm krai, Russia
IEGM 274	Oil-polluted soil, oil-extracting enterprise, Perm region, Russia
IEGM 658	*Taraxacum officinale* rhizosphere, soil, highway, Novosibirsk, Novosibirsk region, Russia
IEGM 659	Soil, highway, Novosibirsk, Novosibirsk region, Russia
IEGM 660	Soil, highway, Novosibirsk, Novosibirsk region, Russia
IEGM 661	Virgin soil, Academgorodok, Novosibirsk, Novosibirsk region, Russia
IEGM 662	Soil, Novosibirsk, Novosibirsk region, Russia
IEGM 695	Water, oilfield area, Perm region, Russia
IEGM 784	Soil, Novosibirsk, Novosibirsk region, Russia
IEGM 785	Virgin soil, Academgorodok, Novosibirsk, Novosibirsk region, Russia
IEGM 786	Soil, Novosibirsk, Novosibirsk region, Russia
IEGM 787	Soil, Novosibirsk, Novosibirsk region, Russia
IEGM 788	Oil-polluted soil, Novosibirsk region, Russia
IEGM 1017	*Dactylis glomerata* rhizosphere, petrol station, Perm, Perm region, Russia
IEGM 1018	*Tussilago farfara* rhizosphere, petrol station, Perm, Perm region, Russia
IEGM 1020	Water, enterprise on production of paracetamol, Perm region, Russia
IEGM 1167	*Polygonum aviculare* rhizosphere, petrol station, Perm, Perm region, Russia
IEGM 1180	Soil, bank of the river Chernaya, Solikamsk, Perm region, Russia
IEGM 1188	Soil, to salt dump, Solikamsk, Perm region, Russia

Table S1 – *Rhodococcus* strains (http://www.iegmcol.ru)

Strain	Isolation source
IEGM 1189	Water, Tyumen region, Russia
IEGM 1204	Soil, Yagel'nyy village, Tyumen region, Russia
IEGM 1220	*Taraxacum officinale* rhizosphere, petrol station, Perm, Perm region, Russia
IEGM 1230	Oil-polluted soil, Perm region, Russia
IEGM 1232	Oil-polluted soil, Perm region, Russia
IEGM 1239	*Euphorbia virgata* rhizosphere, Kungurskiy district, Perm region, Russia
IEGM 1268	Oil-polluted soil, Udmurt Republic, Russia
IEGM 1353	Ni-contaminated soil, metallurgical enterprise area, Perm, Russia
R. qingshengii	
IEGM 247	Soil, lavsan polyethereal fibre production, open joint-stock company “Mogilevkhimvolokno”, Mogilev, Belarus
IEGM 267	Oil-polluted soil oil-extracting enterprise, Perm krai, Russia
IEGM 699	Soil, oilfield outline zone, Perm region, Russia
IEGM 746	Oil-polluted soil, oil-extracting enterprise, Udmurt Republic, Russia
IEGM 1262	Oil slime, Udmurt Republic, Russia
IEGM 1270	Oil slime, Udmurt Republic, Russia
IEGM 1359	Bottom sediment from lake systems, Li Smita Island, Franz Josef Land, Arkhangel'sk region, Russia
R. cerastii	
IEGM 1278	Oil-polluted soil after the remediation, Udmurt Republic, Russia
R. corynebacterioides	
IEGM 931	Soil, *Arctium tomentosum* rhizosphere, petrol station, Perm, Russia
IEGM 1202	coastal soil, the Goltsovoye Lake, the Mammoth Peninsula, Yamalo-Nenets Autonomous Okrug, Tyumen region, Russia
R. fascians	
IEGM 281	Water, oil-extracting enterprise, Perm region, Russia
IEGM 930	*Artemisia vulgaris* rhizosphere, soil, petrol station, Perm, Russia
IEGM 1072	*Artemisia vulgaris* rhizosphere, petrol station site, Perm, Perm region, Russia
IEGM 1158	*Achillea* rhizosphere, soil, former landfill area, Perm, Perm region, Russia
IEGM 1159	*Achillea* rhizosphere, soil, former landfill area, Perm, Perm region, Russia
IEGM 1168	*Artemisia vulgaris* rhizosphere, soil, petrol station, Perm, Perm region, Russia
IEGM 1213	Rhizosphere, soil, Sverdlovsk region, Russia
IEGM 1216	Rhizosphere, soil, Sverdlovsk region, Russia
IEGM 1218	Rhizosphere, soil, Stroganovskiy otval, Sverdlovsk region, Russia
IEGM 1226	Water, Lys'venskiy’s pound, Perm region, Russia
IEGM 1233	*Elytrigia repens* rhizosphere, oilfield, Perm region, Russia
IEGM 1235	*Elytrigia repens* rhizosphere, oilfield, Perm region, Russia
R. globerulus	
IEGM 1019	Soil, Belarus
IEGM 1203	Soil, lake shore Gal'tsovoye, Tyumen region, Russia

Table S1 – *Rhodococcus* strains (http://www.iegmcol.ru)

Strain	Isolation source
R. jostii	
IEGM 489	Active oil well, Belarus
IEGM 1170	*Taraxacum officinale* rhizosphere, gas-pipe area, Perm, Perm region, Russia
IEGM 1193	Oil-polluted soil, biological station, Solikamsk, Perm region, Russia
R. opacus	
IEGM 56	Soil, windbreak, Kherson region, Ukraine
IEGM 57	Oil-polluted soil, Ukraine
IEGM 58	Soil, meadow, Dnepropetrovsk region, Ukraine
IEGM 59	No information
IEGM 246	Soil, lavsan (polyether fibre) production, Belarus
IEGM 248	Soil, lavsan (polyether fibre) production, Belarus
IEGM 249	Soil, lavsan (polyether fibre) production, Belarus
IEGM 261	Soil, lavsan (polyether fibre) production, Belarus
IEGM 262	Soil, lavsan (polyether fibre) production, Belarus
IEGM 263	Soil, lavsan (polyether fibre) production, Belarus
IEGM 264	Soil, lavsan (polyether fibre) production, Belarus
IEGM 488	Oil-polluted water, Un'va river, Perm region, Russia
IEGM 717	Soil, UK
IEGM 765	Oil-polluted soil, Perm region, Russia
IEGM 1157	Soil, Perm, Perm krai, Russia
IEGM 2226	Oil-polluted soil, Perm region, Russia
R. pyridinivorans	
IEGM 1137	Oil-polluted soil, Solikamsk, Perm region, Russia
IEGM 1227	Water, Lys'venskiy's pound, Lys'va, Perm region, Russia
R. rhodochrous	
IEGM 63	Oil-polluted soil, Ukraine
IEGM 64	No information
IEGM 107	Water, the Dnieper river, Dnepropetrovsk region, Ukraine
IEGM 646	Water, Berezniki, Perm krai, Russia
IEGM 757	Oil-polluted soil, Perm region, Russia
IEGM 1138	Oil-polluted soil, Solikamsk, Perm region, Russia
IEGM 1161	*Atriplex* rhizosphere, soil, former landfill area, Perm, Perm region, Russia
IEGM 1162	*Atriplex* rhizosphere, soil, former landfill area, Perm, Perm region, Russia
IEGM 1360	Moss rhizosphere, soil near a stream at hydropost, bukhta Tikhaya, Gukera Island, Franz Josef Land, Arkhangel'sk region, Russia
IEGM 1362	Peat, Pal'tinskoye peat deposit, Perm region, Russia
IEGM 1363	Oil-polluted soil, Udmurt Republic, Russia
R. ruber	
IEGM 90	Surface water reservoir, the Taimir peninsula, Krasnoyarsk region, Russia
IEGM 93	Sandy soil, Irkutsk region, Eastern Siberia, Russia
IEGM 219	Water, the Upper Ilitch river, Russia

Table S1 – *Rhodococcus* strains (http://www.iegmcol.ru)

Strain	Isolation source
IEGM 231	Water, spring, Olkhovski oil-extracting enterprise, Perm krai, Russia
IEGM 232	Soil, Polazna oil-extracting enterprise, Perm region, Russia
IEGM 234	Snow, Polazna oil-extracting enterprise, Perm region, Russia
IEGM 241	Chalk rock (depth 80 m), Gomel region, Belarus
IEGM 320	Mineral water, Surgut district, Tyumen district, Tyumen region, Russia
IEGM 321	Mineral water, Surgut district, Tyumen region, Russia
IEGM 328	Water, well, oil-gas field, Perm krai, Russia
IEGM 334	Sand rock from the depth 6.0 m, Belarus
IEGM 337	Clay from the depth 18 m, Belarus
IEGM 1121	*Plantago* rhizosphere, soil, former landfill area, Perm, Perm region, Russia
IEGM 1122	*Plantago major* rhizosphere, soil, former landfill area, Perm, Perm region, Russia
IEGM 1124	*Atriplex* rhizosphere, soil, former landfill area, Perm, Perm region, Russia
IEGM 1125	*Atriplex* rhizosphere, soil, former landfill area, Perm, Perm region, Russia
IEGM 1126	Soil, *Atriplex* rhizosphere, public green space, Perm, Perm region, Russia
IEGM 1128	*Urtica dioica* rhizosphere, soil, gas-pipe area, Perm, Perm region, Russia
IEGM 1129	*Urtica dioica* rhizosphere, soil, gas-pipe area, Perm, Perm region, Russia
IEGM 1130	*Urtica dioica* rhizosphere, soil, gas-pipe area, Perm, Perm region, Russia
IEGM 1135	*Poa pratensis* rhizosphere, soil, highway to salt dump, Solikamsk, Perm region, Russia
IEGM 1139	*Pimpinella saxifraga* rhizosphere, soil, lakeside, the Gdanski pen., Tyumen region, Russia
IEGM 1141	*Poa* rhizosphere, soil, Sverdlovsk region, Russia
IEGM 1156	*Plantago* rhizosphere, former landfill area, Perm, Perm region, Russia
IEGM 1173	Silt, Suksun's pond, Suksun, Perm region, Russia
IEGM 1210	Rhizosphere, soil, Sverdlovsk region, Russia
IEGM 1211	Rhizosphere, soil, Pokrovskoye village, Sverdlovsk region, Russia
IEGM 1214	Rhizosphere, soil, Pokrovskoye village, Sverdlovsk region, Russia
IEGM 1215	Rhizosphere, soil, Korabel'nyy mys, Sverdlovsk region, Russia
IEGM 1217	Rhizosphere, soil, Sverdlovsk region, Russia
IEGM 1219	Rhizosphere, soil, Sverdlovsk region, Russia
IEGM 1263	Oil-polluted waste, Sosnogorsk, Komi Republic, Russia
IEGM 1352	Ni-contaminated soil, metallurgical enterprise area, Perm, Perm region, Russia
R. wratislaviensis	
IEGM 1171	*Urtica dioica* rhizosphere, soil, petrol station, Perm, Perm region, Russia
R. yunnanensis	
IEGM 1323	Soil, Hooker Island, Franz Josef Land, Arkhangel'sk region, Russia

Table S2 – Growth of *Rhodococcus* strains in the presence of aromatic compounds

Strain	Benzene	Toluene	*o*-Xylene	Naphthalene	Phenol	Pyridine
R. aetherivorans						
IEGM 1250	**Growth**	**Growth**	**Growth**	ND	ND	No growth
R. erythropolis						
IEGM 190	No growth	No growth	No growth	No growth	**Growth**	ND
IEGM 201	No growth	No growth	No growth	No growth	**Growth**	ND
IEGM 202	No growth	No growth	No growth	No growth	No growth	ND
IEGM 203	No growth	No growth	No growth	No growth	No growth	ND
IEGM 204	No growth	No growth	No growth	No growth	No growth	ND
IEGM 212	ND	ND	ND	Growth	ND	ND
IEGM 214	No growth	No growth	No growth	No growth	No growth	ND
IEGM 215	No growth	No growth	No growth	No growth	No growth	ND
IEGM 216	No growth	No growth	No growth	No growth	No growth	ND
IEGM 244	No growth	No growth	No growth	No growth	No growth	ND
IEGM 250	No growth	No growth	No growth	No growth	No growth	ND
IEGM 251	No growth	No growth	No growth	No growth	No growth	ND
IEGM 252	No growth	No growth	No growth	No growth	No growth	ND
IEGM 265	No growth	No growth	No growth	No growth	**Growth**	ND
IEGM 266	ND	ND	ND	**Growth**	ND	ND
IEGM 274	No growth	No growth	No growth	No growth	No growth	ND
IEGM 658	ND	ND	**Growth**	**Growth**	No growth	ND
IEGM 659	ND	ND	**Growth**	**Growth**	No growth	ND
IEGM 660	ND	ND	No growth	**Growth**	No growth	ND
IEGM 661	ND	ND	No growth	**Growth**	No growth	ND
IEGM 662	ND	ND	No growth	**Growth**	No growth	ND
IEGM 695	No growth	No growth	**Growth**	ND	ND	ND
IEGM 784	ND	ND	**Growth**	**Growth**	No growth	ND
IEGM 785	ND	ND	No growth	**Growth**	No growth	ND
IEGM 786	ND	ND	**Growth**	**Growth**	No growth	ND
IEGM 787	ND	ND	No growth	**Growth**	No growth	ND
IEGM 788	ND	ND	**Growth**	**Growth**	**Growth**	ND
IEGM 1017	ND	ND	No growth	No growth	No growth	ND
IEGM 1018	ND	ND	**Growth**	**Growth**	No growth	ND
IEGM 1020	No growth	No growth	**Growth**	ND	ND	ND
IEGM 1167	ND	ND	**Growth**	**Growth**	No growth	ND
IEGM 1180	No growth	No growth	**Growth**	ND	ND	ND
IEGM 1188	No growth	No growth	**Growth**	ND	ND	ND
IEGM 1189	No growth	**Growth**	**Growth**	ND	ND	No growth
IEGM 1204	No growth	No growth	**Growth**	ND	ND	ND
IEGM 1220	No growth	No growth	No growth	**Growth**	No growth	ND
IEGM 1230	Compromised growth	Compromised growth	**Growth**	ND	ND	ND
IEGM 1232	**Growth**	**Growth**	**Growth**	ND	ND	ND
IEGM 1239	No growth	**Growth**	**Growth**	ND	ND	ND
IEGM 1268	No growth	No growth	**Growth**	ND	ND	ND
IEGM 1353	ND	ND	Compromised growth	ND	ND	ND

Table S2 – Growth of *Rhodococcus* strains in the presence of aromatic compounds

Strain	Benzene	Toluene	*o*-Xylene	Naphthalene	Phenol	Pyridine
R. qingshengii						
IEGM 247	Compromised growth	ND	No growth	ND	ND	ND
IEGM 267	ND	ND	Compromised growth	**Growth**	No growth	ND
IEGM 699	No growth	No growth	Compromised growth	ND	ND	ND
IEGM 746	No growth	No growth	**Growth**	ND	ND	ND
IEGM 1262	No growth	**Growth**	**Growth**	ND	ND	ND
IEGM 1270	No growth	No growth	**Growth**	**Growth**	No growth	ND
IEGM 1359	**Growth**	ND	**Growth**	ND	ND	No growth
R. cerastii						
IEGM 1278	No growth	No growth	**Growth**	ND	ND	ND
R. corynebacterioides						
IEGM 931	**Growth**	ND	Compromised growth	No growth	No growth	ND
IEGM 1202	No growth	ND	**Growth**	ND	ND	ND
R. fascians						
IEGM 281	No growth	No growth	No growth	No growth	No growth	ND
IEGM 930	No growth	ND	Compromised growth	No growth	No growth	ND
IEGM 1072	No growth	ND	Compromised growth	**Growth**	No growth	ND
IEGM 1158	No growth	ND	**Growth**	ND	ND	ND
IEGM 1159	No growth	ND	**Growth**	ND	ND	ND
IEGM 1168	No growth	ND	Compromised growth	No growth	No growth	ND
IEGM 1213	No growth	ND	**Growth**	ND	ND	ND
IEGM 1216	No growth	ND	**Growth**	ND	ND	ND
IEGM 1218	No growth	ND	**Growth**	ND	ND	ND
IEGM 1226	No growth	ND	**Growth**	ND	ND	ND
IEGM 1233	**Growth**	**Growth**	**Growth**	ND	ND	ND
IEGM 1235	No growth	**Growth**	**Growth**	ND	ND	ND
R. globerulus						
IEGM 1019	No growth	No growth	Compromised growth	ND	ND	ND
IEGM 1203	No growth	No growth	**Growth**	ND	ND	ND
R. jostii						
IEGM 489	No growth	ND	No growth	ND	ND	ND
IEGM 1170	ND	ND	**Growth**	**Growth**	No growth	ND
IEGM 1193	No growth	ND	**Growth**	ND	ND	No growth
R. opacus						
IEGM 56	Compromised growth	ND	No growth	ND	ND	ND
IEGM 57	**Growth**	ND	No growth	**Growth**	ND	ND
IEGM 58	No growth	ND	No growth	ND	ND	ND
IEGM 59	No growth	ND	No growth	ND	ND	ND
IEGM 246	No growth	ND	No growth	ND	ND	ND

Table S2 – Growth of *Rhodococcus* strains in the presence of aromatic compounds

Strain	Benzene	Toluene	*o*-Xylene	Naphthalene	Phenol	Pyridine
IEGM 248	No growth	ND	No growth	ND	ND	ND
IEGM 249	No growth	ND	No growth	**Growth**	ND	ND
IEGM 261	No growth	ND	No growth	ND	ND	ND
IEGM 262	No growth	ND	No growth	ND	ND	ND
IEGM 263	No growth	ND	No growth	ND	ND	ND
IEGM 264	No growth	ND	No growth	ND	ND	ND
IEGM 488	No growth	ND	No growth	ND	ND	ND
IEGM 717	No growth	ND	No growth	ND	ND	ND
IEGM 765	No growth	ND	No growth	**Growth**	ND	ND
IEGM 1157	No growth	ND	No growth	ND	No growth	ND
IEGM 2226	**Growth**	**Growth**	No growth	**Growth**	ND	ND
R. pyridinivorans						
IEGM 1137	ND	ND	ND	ND	ND	No growth
IEGM 1227	Compromised growth	Compromised growth	Compromised growth	No growth	No growth	ND
R. rhodochrous						
IEGM 63	ND	ND	ND	**Growth**	ND	ND
IEGM 64	ND	ND	ND	**Growth**	ND	ND
IEGM 107	ND	ND	No growth	ND	ND	ND
IEGM 646	ND	ND	ND	**Growth**	ND	ND
IEGM 757	ND	ND	ND	ND	ND	**Growth**
IEGM 1138	**Growth**	ND	**Growth**	ND	ND	No growth
IEGM 1161	ND	ND	No growth	**Growth**	**Growth**	ND
IEGM 1162	ND	ND	**Growth**	**Growth**	**Growth**	ND
IEGM 1360	**Growth**	ND	ND	ND	ND	ND
IEGM 1362	**Growth**	ND	ND	ND	ND	ND
IEGM 1363	**Growth**	ND	ND	ND	ND	ND
R. ruber						
IEGM 90	No growth	No growth	No growth	No growth	No growth	ND
IEGM 93	No growth	**Growth**	No growth	No growth	No growth	ND
IEGM 219	ND	ND	ND	**Growth**	ND	ND
IEGM 231	**Growth**	**Growth**	ND	ND	ND	**Growth**
IEGM 232	No growth	**Growth**	No growth	No growth	No growth	ND
IEGM 234	No growth	**Growth**	No growth	No growth	No growth	ND
IEGM 241	ND	ND	ND	**Growth**	ND	ND
IEGM 320	No growth	No growth	No growth	No growth	No growth	ND
IEGM 321	No growth	No growth	No growth	No growth	No growth	ND
IEGM 328	ND	ND	ND	**Growth**	ND	ND
IEGM 334	No growth	No growth	No growth	**Growth**	No growth	ND
IEGM 337	No growth	No growth	No growth	No growth	No growth	ND
IEGM 1121	**Growth**	**Growth**	**Growth**	**Growth**	Compromised growth	ND
IEGM 1122	Compromised growth	**Growth**	**Growth**	**Growth**	**Growth**	ND
IEGM 1124	**Growth**	**Growth**	ND	**Growth**	No growth	ND
IEGM 1125	Compromised growth	**Growth**	**Growth**	**Growth**	No growth	ND

Table S2 – Growth of *Rhodococcus* strains in the presence of aromatic compounds

Strain	Benzene	Toluene	*o*-Xylene	Naphthalene	Phenol	Pyridine
IEGM 1126	Compromised growth	**Growth**	**Growth**	**Growth**	No growth	ND
IEGM 1128	No growth	ND	**Growth**	ND	ND	ND
IEGM 1129	Compromised growth	**Growth**	**Growth**	**Growth**	No growth	ND
IEGM 1130	No growth	ND	**Growth**	ND	ND	ND
IEGM 1135	No growth	ND	**Growth**	ND	ND	ND
IEGM 1139	No growth	ND	**Growth**	ND	ND	ND
IEGM 1141	No growth	ND	Compromised growth	ND	ND	ND
IEGM 1156	**Growth**	**Growth**	**Growth**	**Growth**	Compromised growth	ND
IEGM 1173	No growth	ND	**Growth**	**Growth**	No growth	ND
IEGM 1210	No growth	ND	**Growth**	ND	ND	ND
IEGM 1211	No growth	ND	**Growth**	ND	ND	ND
IEGM 1214	Compromised growth	ND	**Growth**	ND	ND	ND
IEGM 1215	No growth	ND	**Growth**	ND	ND	ND
IEGM 1217	No growth	ND	**Growth**	ND	ND	ND
IEGM 1219	No growth	ND	**Growth**	ND	ND	ND
IEGM 1263	Compromised growth	ND	**Growth**	ND	ND	No growth
IEGM 1352	ND	ND	No growth	ND	ND	ND
R. wratislaviensis						
IEGM 1171	No growth	ND	Compromised growth	**Growth**	No growth	ND
R. yunnanensis						
IEGM 1323	ND	ND	No growth	ND	ND	ND

Table S3 – Numbers of genes coded for putative enzymes of aromatic compound biodegradation by *Rhodococcus* spp. (a large set of genes is presented)

**Strain**	*R. qingshengii* IEGM 267	*R. qingshengii* IEGM 746	*R. erythropolis* IEGM 1189	*R. qingshengii* IEGM 1359	*R. opacus* IEGM 249	*R. pyridinivorans* IEGM 1137	*R. rhodochrous* IEGM 107	*R. rhodochrous* IEGM 757	*R. rhodochrous* IEGM 1360	*R. ruber* IEGM 231
**Growth substrates**	*o*-Xylene, PAHs	*o*-Xylene	Toluene, *o*-xylene	Benzene, *o*-xylene	Naphthalene	-	-	Pyridine	Benzene	Benzene, toluene, PAHs, aniline, pyridine and its derivatives, coumarin
**Number of genes coded for**										
Dioxygenases (total)	35	30	28	30	69	23	32	41	32	46
catechol 1,2-dioxygenases	1	1	1	1	4	1	2	3	2	2
catechol 2,3-dioxygenases	0	0	0	0	2	1	0	0	0	1
protocatechuate 3,4-dioxygenases	1	1	1	1	1	1	1	1	1	2
protocatechuate 4,5-dioxygenases	0	0	0	0	0	0	0	0	0	0
homoprotocatechuate 2,3-dioxygenase	0	0	0	0	0	0	0	0	0	0
extradiol dioxygenases	2	2	2	2	2	1	2	1	2	6
homogentisate 1,2-dioxygenase	1	2	1	2	1	1	2	2	2	1
gentisate 1,2-dioxygenase	0	0	0	0	3	0	1	2	1	1
benzene 1,2-dioxygenase	0	0	0	0	0	0	0	0	0	1
hydroxyquinol 1,2-dioxygenases	0	0	0	0	0	0	0	0	0	1
2,3-dihydroxy-*p*-cumate-3,4-dioxygenase	0	0	0	0	0	0	0	0	0	0
2,4,5-trihydroxytoluene dioxygenase	0	0	0	0	0	0	0	0	0	0
gallate 4,5-dioxygenase	0	0	0	0	0	0	0	0	0	0
2,3-dihydroxyphenyl propionate1,2-dioxygenase	0	0	0	0	0	0	0	0	0	0
hydroquinone 1,2-dioxygenase	0	0	0	0	0	0	0	0	0	0
3-hydroxyanthranilate 3,4-dioxygenase	1	1	1	1	0	0	0	0	0	0
naphthalene dioxygenase	0	0	0	0	0	0	0	0	0	0
aniline dioxygenases	0	0	0	0	0	0	0	0	0	0
2-aminophenol 1,6-dioxygenase	0	0	0	0	0	0	0	0	0	0

Table S3 – Numbers of genes coded for putative enzymes of aromatic compound biodegradation by *Rhodococcus* spp. (a large set of genes is presented)

**Strain**	*R. qingshengii* IEGM 267	*R. qingshengii* IEGM 746	*R. erythropolis* IEGM 1189	*R. qingshengii* IEGM 1359	*R. opacus* IEGM 249	*R. pyridinivorans* IEGM 1137	*R. rhodochrous* IEGM 107	*R. rhodochrous* IEGM 757	*R. rhodochrous* IEGM 1360	*R. ruber* IEGM 231
**Growth substrates**	*o*-Xylene, PAHs	*o*-Xylene	Toluene, *o*-xylene	Benzene, *o*-xylene	Naphthalene	-	-	Pyridine	Benzene	Benzene, toluene, PAHs, aniline, pyridine and its derivatives, coumarin
**Number of genes coded for**										
2,5-dihydroxypyridine 5,6-dioxygenases	0	0	0	0	0	0	0	0	0	0
phthalate 3,4-dioxygenase	0	0	0	0	1	0	0	0	0	0
terephthalate 1,2-dioxygenase	0	0	0	0	0	0	0	0	0	0
isophthalate 3,4-dioxygenase	0	0	0	0	0	0	0	0	0	0
anthranilate 1,2-dioxygenase reductase	1	1	1	1	1	0	0	0	0	1
3-hydroxyanthranilate 3,4-dioxygenase (EC 1.13.11.6)	1	1	1	1	0	0	0	0	0	0
benzoate 1,2-dioxygenase	1	1	0	0	4	1	2	2	2	1
biphenyl 2,3-dioxygenase	0	0	0	0	0	0	0	0	0	1
indole 3-acetate dioxygenase	0	0	0	0	0	0	0	0	0	0
p-cumate 2,3-dioxygenase	0	0	0	0	0	0	0	0	0	0
4-hydroxyphenyl pyruvate dioxygenase	2	2	2	2	4	2	3	3	3	2
2,4′-dihydroxy acetophenone dioxygenase	0	0	0	0	0	0	0	0	0	0
Monooxygenases (total)	66	66	66	66	97	45	63	74	63	72
flavin-binding monooxygenases	4	3	3	2	3	2	3	4	3	0
benzene/ toluene monooxygenase	0	0	0	0	0	0	0	0	0	0
phenol monooxygenase	0	0	0	0	0	0	0	0	0	3
benzoyl-CoA oxygenases/ reductases	0	0	0	0	1	0	1	0	1	0

Table S3 – Numbers of genes coded for putative enzymes of aromatic compound biodegradation by *Rhodococcus* spp. (a large set of genes is presented)

**Strain**	*R. qingshengii* IEGM 267	*R. qingshengii* IEGM 746	*R. erythropolis* IEGM 1189	*R. qingshengii* IEGM 1359	*R. opacus* IEGM 249	*R. pyridinivorans* IEGM 1137	*R. rhodochrous* IEGM 107	*R. rhodochrous* IEGM 757	*R. rhodochrous* IEGM 1360	*R. ruber* IEGM 231
**Growth substrates**	*o*-Xylene, PAHs	*o*-Xylene	Toluene, *o*-xylene	Benzene, *o*-xylene	Naphthalene	-	-	Pyridine	Benzene	Benzene, toluene, PAHs, aniline, pyridine and its derivatives, coumarin
**Number of genes coded for**										
phenylacetyl-CoA oxygenases/ reductases, or 1,2-phenylacetyl-CoA epoxidases PaaE/PaaJ/ PaaK (EC 1.14.13.149)	1	1	1	1	0	0	0	0	0	0
4-hydroxybenzoate 3-hydroxylase	1	1	1	1	4	2	3	3	3	1
3-hydroxybenzoate 4-hydroxylase	0	0	0	0	0	0	0	0	0	0
3-hydroxybenzoate 6-hydroxylase	0	0	0	0	0	0	0	0	0	1
salicylate 1-hydroxylase	0	0	1	1	0	0	1	0	1	0
salicylate-5-hydroxylase	0	0	0	0	0	0	0	0	0	0
3-hydroxyphenyl propionate 2-hydroxylase	0	0	0	0	0	0	0	0	0	0
3-hydroxyphenyl acetate 6-hydroxylase	0	0	0	0	0	0	0	0	0	0
4-hydroxyphenyl acetate 3-hydroxylase	1	1	1	1	7	2	1	1	1	0
resorcinol 4-hydroxylase	0	0	0	0	0	0	0	0	0	0
p-cresol methylhydroxylase	0	0	0	0	0	0	0	0	0	0
2-aminobenzoyl-CoA monooxygenase/ reductase	0	0	0	0	0	0	0	0	0	0
3-(3-hydroxyphenyl)propionate hydroxylase	0	0	0	0	0	0	0	0	0	0
6-hydroxynicotinate 3-monooxygenase	0	0	0	0	0	0	0	0	0	0
Multicopper polyphenol oxidases	1	1	1	1	1	1	1	1	1	4

Table S3 – Numbers of genes coded for putative enzymes of aromatic compound biodegradation by *Rhodococcus* spp. (a large set of genes is presented)

**Strain**	*R. qingshengii* IEGM 267	*R. qingshengii* IEGM 746	*R. erythropolis* IEGM 1189	*R. qingshengii* IEGM 1359	*R. opacus* IEGM 249	*R. pyridinivorans* IEGM 1137	*R. rhodochrous* IEGM 107	*R. rhodochrous* IEGM 757	*R. rhodochrous* IEGM 1360	*R. ruber* IEGM 231
**Growth substrates**	*o*-Xylene, PAHs	*o*-Xylene	Toluene, *o*-xylene	Benzene, *o*-xylene	Naphthalene	-	-	Pyridine	Benzene	Benzene, toluene, PAHs, aniline, pyridine and its derivatives, coumarin
**Number of genes coded for**										
Dehydrogenases (total)	387	367	350	348	731	256	352	429	348	288
nicotinate dehydrogenase	0	0	0	0	0	0	0	0	0	0
aldehyde dehydrogenase	13	13	11	12	41	6	15	20	15	5
succinate-semialdehyde dehydrogenase	1	1	1	1	5	1	1	0	1	1
3,4-dihydroxy-3,4-dihydrophthalate dehydrogenase	0	0	0	0	0	0	0	0	0	0
4,5-dihydro-4,5-dihydroxyphthalate dehydrogenase	0	0	0	0	0	0	0	0	0	0
Hydrolases	125	116	116	116	229	96	115	133	115	123
phenol hydrolase	0	0	0	0	1	1	0	0	0	0
N-formylmaleamate deformylase	0	0	0	0	0	0	0	0	0	0
maleamate amidohydrolase	0	0	0	0	0	0	0	0	0	1
amidase	2	2	2	2	2	0	0	0	0	3
formamidase	1	1	1	1	1	1	1	1	1	0
fumarylacetoacetate hydrolase family	1	1	0	1	6	0	2	2	2	0
hydroxymuconic semialdehyde hydrolase	3	2	2	2	0	1	2	2	2	1
vanillate 3-O demethylase	4	2	2	4	20	4	7	8	7	5
isovanillate 4-O demethylase	0	0	0	0	0	0	0	0	0	0
Maleate isomerase	0	0	1	0	2	0	0	0	0	0
Catalases	4	3	3	4	8	4	5	4	5	3
Lipases	27	21	24	20	31	12	21	23	21	22
Nitrate reductase	1	1	1	1	3	3	3	4	3	3
Nitrite reductase	1	1	1	1	2	1	1	1	1	3
4,5-Dihydroxyphthalate decarboxylase	0	0	0	0	0	0	0	0	0	0
